# Buffalo Medical and Surgical Association

**Published:** 1885-02

**Authors:** Clayton M. Daniels

**Affiliations:** Secretary pro tem.


### Society Reports.

### Buffalo Medical and Surgical Association.

*Regular Meeting, Dec. 2, 1884.*

President, Dr. F. W. Bartlett, in the Chair.

Present—Drs. Hubbell, Cronyn, Macniel, Van Peyma, Wetmore, Hartwig, Runner, Weil, Coakley, Brecht, Hawkins, Stockton, Frederick, Park, Barnes, and persons.

In the absence of Dr. Peterson, Dr. Daniels was elected Secretary *pro tem.*

Under the head of "Reading and Discussion of Essay," Dr. Hartwig, the essayist for the evening, stated that he was hardly prepared, on account of the limited time that he had had to prepare his paper, and suggested that further time be allowed, but that if the association desired, he could give it in the form of a lecture from his notes, if he would be permitted to subsequently put it in proper shape for publication.

Upon a motion being put, he was unanimously requested to proceed. (The paper will appear in a future number of the Journal, entitled, "Atypical Case of Pneumonia.")

*Discussion*—Dr. Van Peyma said that the points made seemed to be more in the disagreement in classification of various authors. Also, that as for himself, he considered that the "cheesy" was a second stage of the "catarrhal" form. Tubercular disease he did not believe to be an entity, any more so than in other or all diseases. He did not believe in entities.

Dr. Cronyn said that the essayist had left very little tangible to discuss. He expected to hear the report of a typical case of pneumonia, which is rare. In the disease, if resolution does not occur within a reasonable time, something else does, and we may have many forms, and all the usual sequences, of chronic disease of the lungs. He did not wish to discuss the micro-organism nonsense, and felt that he had a right to differ from some of the authors. Dr. Flint had called it pneumonic fever, and gave large doses of quinine; also said that it was wise to consider separately the sthenic and asthenic forms.

Dr. Bartlett said that he thought a typical case would be described. He thought that in daily practice we should carefully differentiate tuberculosis. Believed, with Dr. Cronyn, that it was quite proper to differentiate the sthenic from the asthenic form. Thought it probable that the paper would come up for future discussion.

Dr. Hartwig, in closing, said that he expected to hear more from the older members. In replying to Dr. Van Peyma, he maintained that the "cheesy" had a definite form, destructive in and between vesicles, and should be properly so recognized. Thought it wrong to name anything else "cheesy." He admitted that other forms may look similar, but in reality were not of this distinctive form, as the true "cheesy" consisted of broken down interstitial tissue, and we should give it a nomenclature, and not confound it with phthisis or anything else.

Dr. Van Peyma further stated his belief that the "cheesy" form was only a secondary stage of the croupous variety.

Dr. Hartwig remarked that evidently some members were misled by the title of his paper, considering "Atypical" a misprint, intending to be two words, but that it was correct.

Dr. Bartlett said that with that explanation it would literally mean "irregular," and would explain why some were expecting to hear the report of "a typical case."

*Prevailing Diseases*—Rheumatism, scarlatina, diphtheria, sore throat of various kinds, and Dr. Bartlett had noted that the sequelæ of measles was unusually obstinate.

*Reports of Committees*—Dr. Hartwig said that the doctors who had been asked to report concerning midwives were somewhat dilatory, and asked that his committee be continued, which, upon motion, was granted.

The President stated that our Secretary, Dr. Peterson, had left the city to assume professional duties elsewhere, and that we were likely to be without a Secretary, and asked what action should be taken. He further stated that Dr. Peterson had not, as yet, formally resigned.

Upon the suggestion of Dr. Cronyn, it was decided to wait until Dr. Peterson be communicated with before formally filling the vacancy.

Adjourned.

Clayton M. Daniels, *Secretary pro tem.*

